# A wireless body area network of intelligent motion sensors for computer assisted physical rehabilitation

**DOI:** 10.1186/1743-0003-2-6

**Published:** 2005-03-01

**Authors:** Emil Jovanov, Aleksandar Milenkovic, Chris Otto, Piet C de Groen

**Affiliations:** 1 Electrical and Computer Engineering Department, University of Alabama in Huntsville, Huntsville, Alabama 35899, USA; 2 Division of Biomedical Informatics, Mayo Clinic College of Medicine, Rochester, Minnesota 55905, USA

## Abstract

**Background:**

Recent technological advances in integrated circuits, wireless communications, and physiological sensing allow miniature, lightweight, ultra-low power, intelligent monitoring devices. A number of these devices can be integrated into a Wireless Body Area Network (WBAN), a new enabling technology for health monitoring.

**Methods:**

Using off-the-shelf wireless sensors we designed a prototype WBAN which features a standard ZigBee compliant radio and a common set of physiological, kinetic, and environmental sensors.

**Results:**

We introduce a multi-tier telemedicine system and describe how we optimized our prototype WBAN implementation for computer-assisted physical rehabilitation applications and ambulatory monitoring. The system performs real-time analysis of sensors' data, provides guidance and feedback to the user, and can generate warnings based on the user's state, level of activity, and environmental conditions. In addition, all recorded information can be transferred to medical servers via the Internet and seamlessly integrated into the user's electronic medical record and research databases.

**Conclusion:**

WBANs promise inexpensive, unobtrusive, and unsupervised ambulatory monitoring during normal daily activities for prolonged periods of time. To make this technology ubiquitous and affordable, a number of challenging issues should be resolved, such as system design, configuration and customization, seamless integration, standardization, further utilization of common off-the-shelf components, security and privacy, and social issues.

## Introduction

Wearable health monitoring systems integrated into a telemedicine system are novel information technology that will be able to support early detection of abnormal conditions and prevention of its serious consequences [1,2]. Many patients can benefit from continuous monitoring as a part of a diagnostic procedure, optimal maintenance of a chronic condition or during supervised recovery from an acute event or surgical procedure.

Important limitations for wider acceptance of the existing systems for continuous monitoring are: a) unwieldy wires between sensors and a processing unit, b) lack of system integration of individual sensors, c) interference on a wireless communication channel shared by multiple devices, and d) nonexistent support for massive data collection and knowledge discovery. Traditionally, personal medical monitoring systems, such as Holter monitors, have been used only to collect data for off-line processing. Systems with multiple sensors for physical rehabilitation feature unwieldy wires between electrodes and the monitoring system. These wires may limit the patient's activity and level of comfort and thus negatively influence the measured results. A wearable health-monitoring device using a Personal Area Network (PAN) or Body Area Network (BAN) can be integrated into a user's clothing [3]. This system organization, however, is unsuitable for lengthy, continuous monitoring, particularly during normal activity [4], intensive training or computer-assisted rehabilitation [5]. Recent technology advances in wireless networking [6], micro-fabrication [7], and integration of physical sensors, embedded microcontrollers and radio interfaces on a single chip [8], promise a new generation of wireless sensors suitable for many applications [9]. However, the existing telemetric devices either use wireless communication channels exclusively to transfer raw data from sensors to the monitoring station, or use standard high-level wireless protocols such as Bluetooth that are too complex, power demanding, and prone to interference by other devices operating in the same frequency range. These characteristics limit their use for prolonged wearable monitoring. Simple, accurate means of monitoring daily activities outside of the laboratory are not available [12]; at the present, only estimates can be obtained from questionnaires, measures of heart rate, video assessment, and use of pedometers [13] or accelerometers [14]. Finally, records from individual monitoring sessions are rarely integrated into research databases that would provide support for data mining and knowledge discovery relevant to specific conditions and patient categories.

Increased system processing power allows sophisticated real-time data processing within the confines of the wearable system. As a result, such wearable system can support biofeedback and generation of warnings. The use of biofeedback techniques has gained increased attention among researchers in the field of physical medicine and tele-rehabilitation [5]. Intensive practice schedules have been shown to be important for recovery of motor function [22]. Unfortunately, an aggressive approach to rehabilitation involving extensive therapist-supervised motor training is not a realistic expectation in today's health care system where individuals are typically seen as outpatients about twice a week for no longer than 30–45 min. Wearable technology and biofeedback systems appear to be a valid alternative, as they reduce the extensive time to set-up a patient before each session and require limited time involvement of physicians and therapists. Furthermore, wearable technology could potentially address a second factor that hinders enthusiasm for rehabilitation, namely the fact that setting up a patient for the procedure is rather time-consuming. This is because tethered sensors need to be positioned on the subject, attached to the equipment, and a software application needs to be started before each session. Wearable technology allows sensors that will be positioned on the subject for prolonged periods, therefore eliminating the need to position them for every training session. Instead, a personal server such as a PDA can almost instantly initiate a new training session whenever the subject is ready and willing to exercise. In addition to home rehabilitation, this setting also may be beneficial in the clinical setting, where precious time of physicians and therapists could be saved. Moreover, the system can issue timely warnings or alarms to the patient, or to a specialized medical response service in the event of significant deviations of the norm or medical emergencies. However, as for all systems, regular, routine maintenance (verifying configuration and thresholds) by a specialist is required.

Typical examples of possible applications include stroke rehabilitation, physical rehabilitation after hip or knee surgeries, myocardial infarction rehabilitation, and traumatic brain injury rehabilitation. The assessment of the effectiveness of rehabilitation procedures has been limited to the laboratory setting; relatively little is known about rehabilitation in real-life situations. Miniature, wireless, wearable technology offers a tremendous opportunity to address this issue.

We propose a wireless BAN composed of off-the-shelf sensor platforms with application-specific signal conditioning modules [10]. In this paper, we present a general system architecture and describe a recently developed activity sensor "ActiS". ActiS is based on a standard wireless sensor platform and a custom sensor board with a one-channel bio amplifier and two accelerometers [11]. As a heart sensor, ActiS can be used to monitor heart activity and position of the upper trunk. The same sensor can be used to monitor position and activity of upper and lower extremities. A wearable system with ActiS sensors would also allow one to assess metabolic rate and cumulative energy expenditure as a valuable parameter in the management of many medical conditions. An early version of the ActiS has been based on a custom developed wireless intelligent sensor and custom wireless protocols in the license-free 900 MHz Scientific and Medical Instruments (ISM) band [15]. Our initial experience indicated the importance of standard sensor platforms with ample processing power, minute power consumption, and standard software support. Such platforms were not available on the market during the design of our first prototype system. The recent introduction of an IEEE standard for low-power personal area networks (802.15.4) and ZigBee protocol stack [16], as well as new ZigBee compliant Telos sensor platform [17], motivated the development of the new system presented in this paper. TinyOS support for the selected sensor platform facilitates rapid application development [18]. Standard hardware and software architecture facilitate interoperable systems and devices that are expected to significantly influence next generation health systems [19]. This trend can also be observed in recently developed physiological monitors systems from Harvard [20] and Welch-Allen [21].

## System Architecture

**Figure 1:**
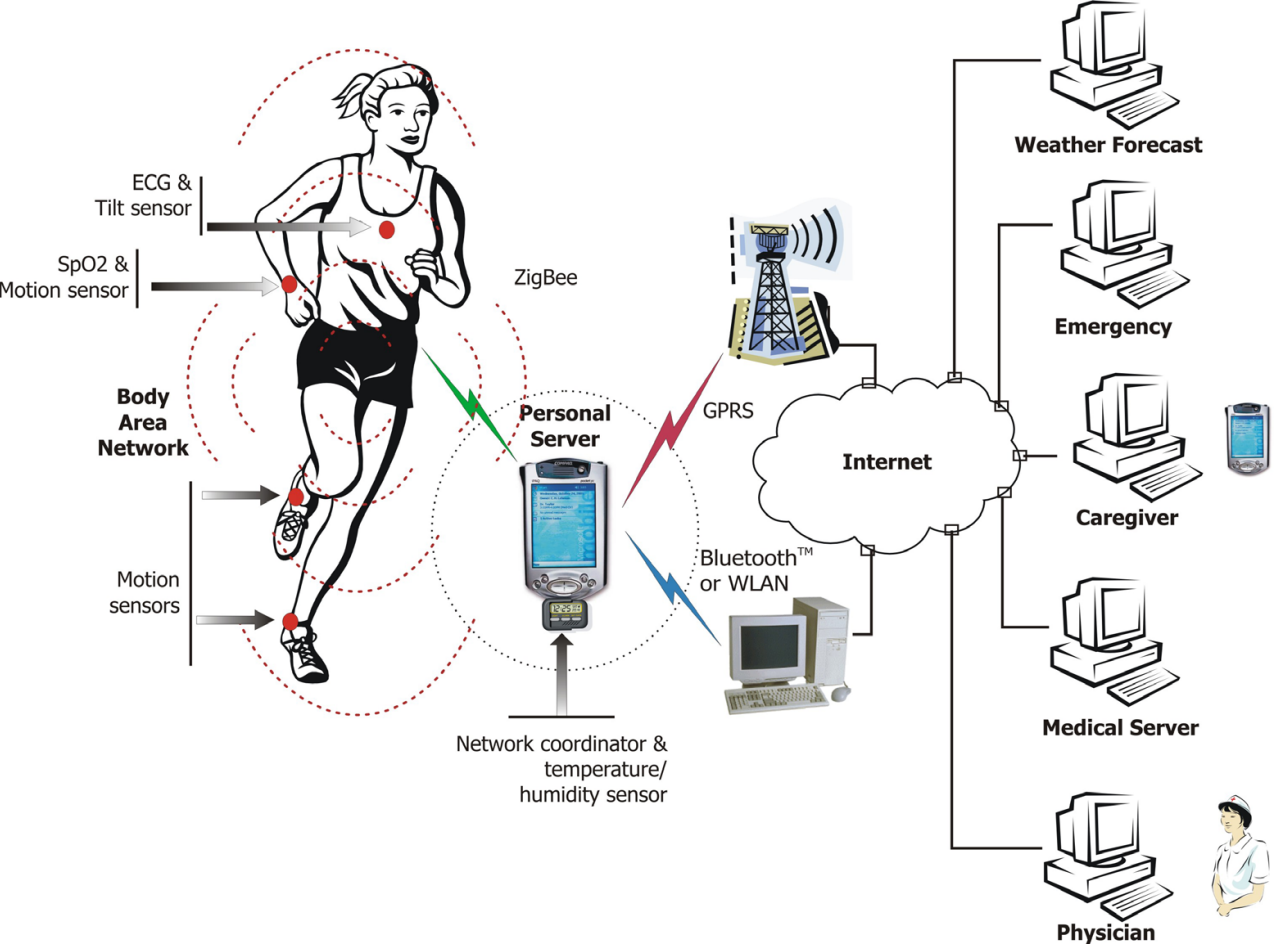
Wireless Body Area Network of Intelligent Sensors for Patient Monitoring

**Figure 2:**
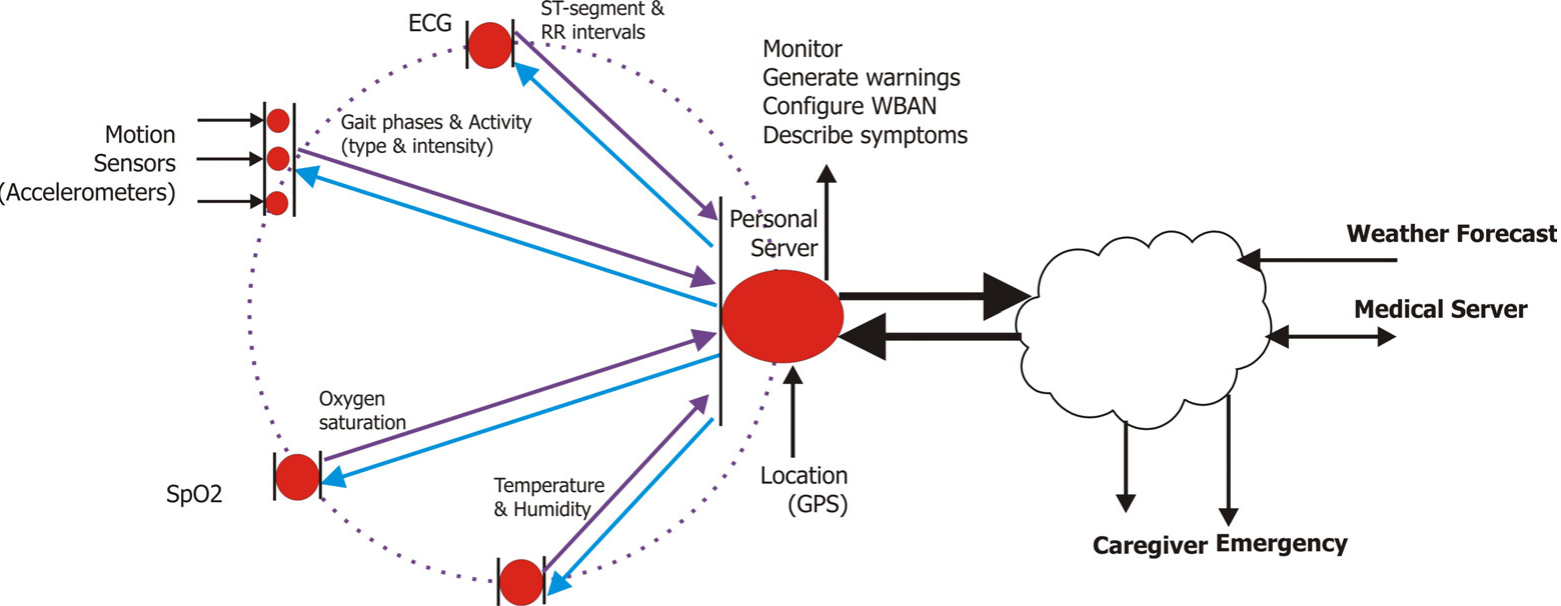
Data flow in an integrated WWBAN

Continuous technological advances in integrated circuits, wireless communication, and sensors enable development of miniature, non-invasive physiological sensors that communicate wirelessly with a personal server and subsequently through the Internet with a remote emergency, weather forecast or medical database server; using baseline (medical database), sensor (WBAN) and environmental (emergency or weather forecast) information, algorithms may result in patient-specific recommendations. The personal server, running on a PDA or a 3 G cell phone, provides the human-computer interface and communicates with the remote server(s). Figure 1 shows a generalized overview of a multi-tier system architecture; the lowest level encompasses a set of intelligent physiological sensors; the second level is the personal server (Internet enabled PDA, cell-phone, or home computer); and the third level encompasses a network of remote health care servers and related services (Caregiver, Physician, Clinic, Emergency, Weather). Each level represents a fairly complex subsystem with a local hierarchy employed to ensure efficiency, portability, security, and reduced cost. Figure 2 illustrates an example of information flow in an integrated WBAN system.

### Sensor level

A WBAN can include a number of physiological sensors depending on the end-user application. Information of several sensors can be combined to generate new information such as total energy expenditure. An extensive set of physiological sensors may include the following:

• an ECG (electrocardiogram) sensor for monitoring heart activity

• an EMG (electromyography) sensor for monitoring muscle activity

• an EEG (electroencephalography) sensor for monitoring brain electrical activity

• a blood pressure sensor

• a tilt sensor for monitoring trunk position

• a breathing sensor for monitoring respiration

• movement sensors used to estimate user's activity

• a "smart sock" sensor or a sensor equipped shoe insole used to delineate phases of individual steps

These physiological sensors typically generate analog signals that are interfaced to standard wireless network platforms that provide computational, storage, and communication capabilities. Multiple physiological sensors can share a single wireless network node. In addition, physiological sensors can be interfaced with an intelligent sensor board that provides on-sensor processing capability and communicates with a standard wireless network platform through serial interfaces.

The wireless sensor nodes should satisfy the following requirements: minimal weight, miniature form-factor, low-power operation to permit prolonged continuous monitoring, seamless integration into a WBAN, standard-based interface protocols, and patient-specific calibration, tuning, and customization. These requirements represent a challenging task, but we believe a crucial one if we want to move beyond 'stovepipe' systems in healthcare where one vendor creates all components. Only hybrid systems implemented by combining off-the-shelf, commodity hardware and software components, manufactured by different vendors promise proliferation and dramatic cost reduction.

The wireless network nodes can be implemented as tiny patches or incorporated into clothes or shoes. The network nodes continuously collect and process raw information, store them locally, and send them to the personal server. Type and nature of a healthcare application will determine the frequency of relevant events (sampling, processing, storing, and communicating). Ideally, sensors periodically transmit their status and events, therefore significantly reducing power consumption and extending battery life. When local analysis of data is inconclusive or indicates an emergency situation, the upper level in the hierarchy can issue a request to transfer raw signals to the upper levels where advanced processing and storage is available.

### Personal server level

The personal server performs the following tasks:

• Initialization, configuration, and synchronization of WBAN nodes

• Control and monitor operation of WBAN nodes

• Collection of sensor readings from physiological sensors

• Processing and integration of data from various physiological sensors providing better insight into the users state

• Providing an audio and graphical user-interface that can be used to relay early warnings or guidance (e.g., during rehabilitation)

• Secure communication with remote healthcare provider servers in the upper level using Internet services

The personal server can be implemented on an off-the-shelf Internet-enabled PDA (Personal Digital Assistant) or 3 G cell phone, or on a home personal computer. Multiple configurations are possible depending on the type of wireless network employed. For example, the personal server can communicate with individual WBAN nodes using the Zigbee wireless protocol that provides low-power network operation and supports virtually an unlimited number of network nodes. A network coordinator, attached to the personal server, can perform some of the pre-processing and synchronization tasks. Other communication scenarios are also possible. For example, the personal server running on a Bluetooth or WLAN enabled PDA can communicate with remote upper-level services through a home computer; the computer then serves as a gateway (Figure 1).

Relying on off-the-shelf mobile computing platforms is crucial, as these platforms will continue to grow in their capabilities and quality of services. The challenging tasks are to develop robust applications that provide simple and intuitive services (WBAN setup, data fusion, questionnaires describing detailed symptoms, activities, secure and reliable communication with remote medical servers, etc). Total information integration will allow patients to receive directions from their healthcare providers based on their current conditions.

### Medical services

We envision various medical services in the top level of the tiered hierarchy. A healthcare provider runs a service that automatically collects data from individual patients, integrates the data into a patient's medical record, processes them, and issues recommendations, if necessary. These recommendations are also documented in the electronic medical record. If the received data are out of range or indicate an imminent medical condition, an emergency service can be notified (this can also be done locally at the personal server level). The exact location of the patient can be determined based on the Internet access entry point or directly if the personal server is equipped with a GPS sensor. Medical professionals can monitor the activity of the patient and issue altered guidance based on the new information, other prior known and relevant patient data, and the patient's environment (e.g., location and weather conditions).

The large amount of data collected through such services will allow quantitative analysis of various conditions and patterns. For example, suggested targets for stride and forces of hip replacement patients could be suggested according to the previous history, external temperature, time of the day, gender, and current physiological parameters (e.g., heart rate). Moreover, the results could be stored in research databases that will allow researchers to quantify the contribution of each parameter to a given condition if adequate numbers of patients are studied in this manner. Again, it is important to emphasize that the proposed approach requires seamless integration of large amounts of data into a research database in order to be able to perform meaningful statistical analyses.

## ActiS – Activity Sensor

**Figure 3:**
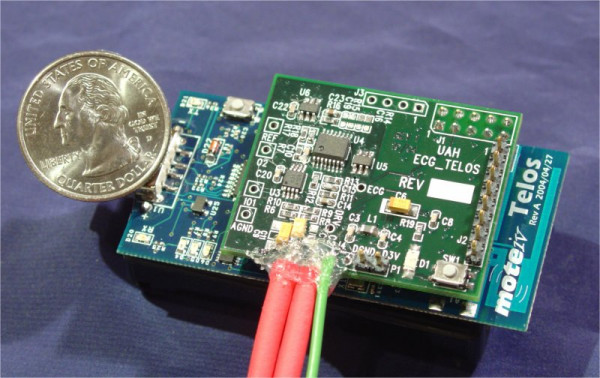
Telos wireless platform with intelligent signal processing daughtercard ISPM

**Figure 4:**
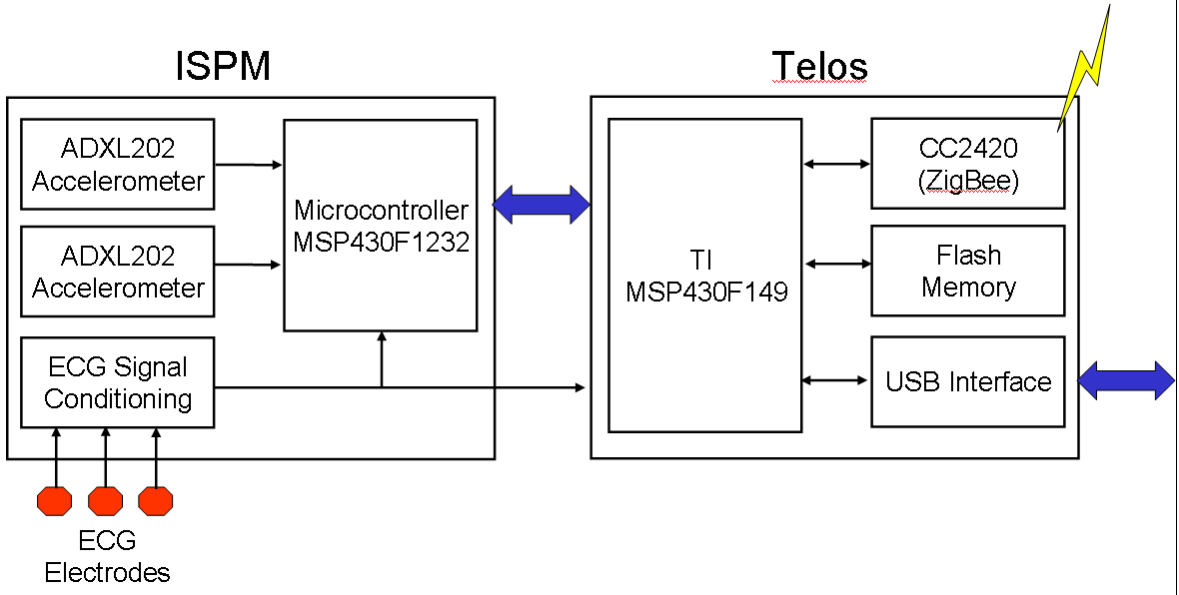
Block diagram of the activity sensor (Telos platform and ISPM module)

The ActiS sensor was developed specifically for WBAN-based, wearable computer-assisted, rehabilitation applications. With this concept in mind, we integrated a one-channel bio-amplifier and three accelerometer channels with a low power microcontroller into an intelligent signal processing board that can be used as an extension of a standard wireless sensor platform. ActiS consists of a standard sensor platform, Telos, from Moteiv and a custom Intelligent Signal Processing Module – ISPM (Figure 3). A block diagram of the sensor node is shown in Figure 4.

The Telos platform is an ideal fit for this application due to small footprint and open source system software support. A second generation of the Telos platform features an 8 MHz MSP430F1611 microcontroller with integrated 10 KB of RAM and 48 KB of flash memory, a USB (Universal Serial Bus) interface for programming and communication, and an integrated wireless ZigBee compliant radio with on-board antenna [11]. In addition, the Telos platform includes humidity, temperature, and light sensors that could be used as ambient sensors. The Telos platform features a 10-pin expansion connector that allows one UART (Universal Asynchronous Receiver Transmitter) and I2 C interface, two general-purpose I/O lines, and three analog input lines.

The ISPM extends the capabilities of Telos by adding two perpendicular dual axis accelerometers (Analog Devices ADXL202) and a bio-amplifier with a signal conditioning circuit. The ISPM has its own MSP430F1232 processor for sampling and low-level data processing. This microcontroller was selected primarily for its compact size and ultra low power operation. Other features that were desirable for this design were the 10-bit ADC and the timer capture/compare registers that are used for acquisition of data from the accelerometers. The F1232 has hardware UART that is used for communications with Telos.

The ISPM's two ADXL202 accelerometers cover all three axes of motion. One ADXL202 is mounted directly on the ISPM board and collects data for the X and Y axes in the same plane. The second ADXL202 is mounted on a daughter card that extends vertically from the ISPM.

The user's physiological state is monitored using an on-board bio-amplifier implemented using an instrumentation amplifier with a signal conditioning circuit. The bio-amplifier could be used for electromyogram (EMG) or electrocardiogram (ECG) monitoring. The output of the signal conditioning circuit is connected to the local microcontroller as well as to the microcontroller on the Telos board via the expansion connector. The AD converter on the Telos board has a higher resolution (12 bit) than the F1232 on the ISPM (10 bit). This configuration gives flexibility of utilizing either microcontroller to process physiological signals.

**Figure 5:**
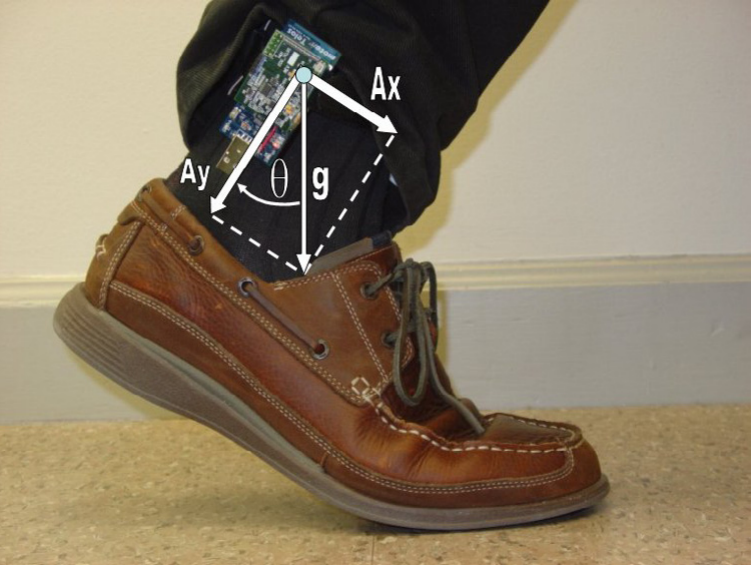
Activity sensor on an ankle with symbolic representation of acceleration components

**Figure 6:**
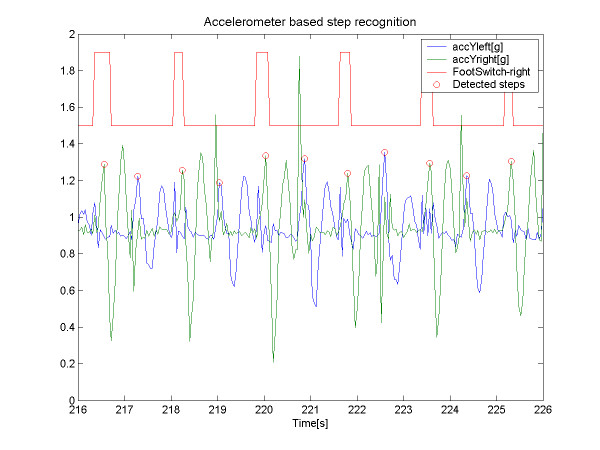
Accelerometer based step detection using ankle sensors

An example application of the ActiS sensor as motion sensor on an ankle is given in Figure 5. This figure also visualizes the main components of acceleration during slow movements as projections of the gravity force (g) on the accelerometer's reference axes – A_x _and A_y_. Rotations of the sensor in the vertical plane (Θ) can be estimated as Θ = arctan(A_x _/ A_y_). A compensation for non-ideal vertical placement can be achieved using the second accelerometer (not mounted in this photo) at 90-degree angle. Instead of calculating the angular position, many systems use off-the-shelf gyroscopes to measure angular velocity for the detection of gait phases [32]. A typical example of step detection is illustrated in Figure 6.

## Issues and Applications

WBAN systems can capitalize on recent technological advances that have enabled new methods for studying human activity and motion, making extended activity analysis more feasible. However, before WBAN becomes a widely accepted concept, a number of challenging *system design *and *social issues *should be resolved. If resolved successfully, WBAN systems will open a whole range of possible new applications that can significantly influence our lives.

### System Design Issues

The development of pedometers and Micro-ElectroMechanical Systems (MEMS) accelerometers and gyroscopes show great promise in the design of wearable sensors. The main system design issues include:

• types of sensors

• power source

• size and weight of sensors

• wireless communication range and transmission characteristics of wearable sensors

• sensor location and mounting

• seamless system configuration

• automatic uploads to the patient's electronic medical record

• intuitive and simple user interface

#### Types of sensors

As for sensors, accelerometers and gyroscopes offer greater sensitivity and are more applicable for monitoring of motion since they generate continuous output. Bouten *et al *[27] found that frequency of human induced activity ranges from 1 to 18 Hz. Sampling rates in the existing projects vary from 10 – 100 Hz. Almost all projects in the last five years use MEMS accelerometers or a combination of accelerometers and gyroscopes [34,35]. As examples of full sets of sensors for research purposes, "MIThril" and Shoe Integrated Gait Sensor (SIGS) [26] systems feature 3 axes of gyroscopes, 3 axes of accelerometers, two piezoelectric sensors, two electric field sensors, two resistive band sensors, and four force sensitive resistors. These sensors can be mounted on the back of a shoe and in a shoe insole, respectively. Researchers at University of Washington School of Nursing have used off-the-shelf tri-axis accelerometer modules to study physical movement in COPD (Chronic Obstructive Pulmonary Disease) patients [2]. Both Lancaster University, UK, and ETH Zurich, Switzerland, have developed custom hardware realizing arrays of inertial sensor networks [24]. Lancaster used an array of 30 two-axis accelerometers. Similarly, ETH Zurich used a modular harness design [25].

The majority of foot-contact pedometers are designed to count steps only. Although they have been studied for use in complex energy estimation and have even shown a high degree of accuracy for walking / running activities [2] they are not well suited for rehabilitation.

#### Power source, size/weight, and transmission characteristics

To be unobtrusive, the sensors must be lightweight with small form factor. The size and weight of sensors is predominantly determined by the size and weight of batteries. Requirements for extended battery life directly oppose the requirement for small form factor and low weight. This implies that sensors have to be extremely power efficient, as frequent battery changes for multiple WBAN sensors would likely hamper users' acceptance and increase the cost. In addition, low power consumption is very important as we move toward future generations of implantable sensors that would ideally be self-powered, using energy extracted from the environment.

The radio communication poses the most significant energy consumption problem. Intelligent on-sensor signal processing has the potential to save power by transmitting the processed data rather than raw signals, and consequently to extend battery life. A careful trade-off between communication and computation is crucial for an optimal design. It appears that the most promising wireless standard for WBAN applications is ZigBee, as it represents an emerging wireless technology for the low-power, short-range, wireless sensors.

#### Location of Sensors

Although the purpose of the measurement does influence sensor location, researchers seem to disagree on the ideal body location for sensors. A motion sensor attached to an ankle is the most discriminative single position for state recognition, while a combination of hip and ankle sensors discriminates the states even more [25]. In a study of the relationship between metabolic energy expenditure and various activities, researchers at Eindhoven University of Technology, the Netherlands, placed tri-axial accelerometers on a subject's back waistline [27]. Krause *et al *use two accelerometers on the SenseWear armband [31]. Lee *et al *[2] placed accelerometer sensors in the subject's thigh pocket in order to measure angular position and velocity of the thigh. Doing so, they were able to accurately monitor a subject's activity and with the assistance of gyroscopes and compass headings were able to successfully estimate a subject's change in location. Some systems employ large arrays of wearable sensors. Laerhoven *et al *developed a loose fitting lab coat and trousers [24] consisting of 30 sensors; Kern *et al *[25]developed tighter fitting modular harnesses including a total of 48 sensors. Sensor attachment is also a critical factor, since the movement of loosely attached sensors creates spurious oscillations after an abrupt movement that can generate false events or mask real events.

#### Seamless system configuration

The intelligent WBAN sensors should allow users to easily assemble a robust ad-hoc WBAN, depending on the user's state of health. We can imagine standard off-the-shelf sensors, manufactured by different vendors, and sold "over-the-counter" [19]. Each sensor should be able to identify itself and declare its operational range and functionality. In addition, they should support easy customization for a given application.

#### Algorithms

Application-specific algorithms mostly use digital signal pre-processing combined with a variety of artificial intelligence techniques to model user's states and activity in each state. Digital signal processing include filters to resolve high and low frequency components of a signal, wavelet transform algorithms to correlate heel-strike and toe-off (steps) to angular velocity measured via gyroscopes [30], power spectrum analysis and a Gaussian model to classify activity types [26]. Artificial intelligence techniques may include fuzzy logic [28] and Kohonen self-organizing maps [31]. Some systems use physiological signals to improve context identification [31]. It has been shown that the activity-induced energy expenditure (AEE) is well correlated with the sum of integrals of the high frequency component of each individual axis [27].

Most of the algorithms in the open literature are not executed in real-time, or require powerful computing platforms such as laptops for real-time analysis.

### Social Issues

Social issues of WBAN systems include privacy/security and legal issues. Due to communication of health-related information between sensors and servers, all communication over WBAN and Internet should be encrypted to protect user's privacy. Legal regulation will be necessary to regulate access to patient-identifiable information.

### Possible applications

The WBAN technology can be used for computer-assisted physical rehabilitation in ambulatory settings and monitoring of trends during recovery. An integrated system can synergize the information from multiple sensors, warn the user in the case of emergencies, and provide feedback during supervised recovery or normal activity. Candidate applications include post-stroke rehabilitation, orthopaedic rehabilitation (e.g. hip/knee replacement rehabilitation), and supervised recovery of cardiac patients [36]. In the case of orthopaedic rehabilitation the system can measure forces and accelerations at different points and provide feedback to the user in real-time. Unobtrusive monitoring of cardiac patients can be used to estimate intensity of activities in user's daily routine and correlate it with the heart activity.

In addition, WBAN systems can be used for gait phase detection during programmable, functional electrical stimulation [33], analysis of balance and monitoring of Parkinson's disease patients in the ambulatory setting [32], computer supervision of health and activity status of elderly, weight loss therapy, obesity prevention, or in general promotion of a healthy, physically active, lifestyle.

## Conclusion

A wearable Wireless Body Area Network (WBAN) of physiological sensors integrated into a telemedical system holds the promise to become a key infrastructure element in remotely supervised, home-based patient rehabilitation. It has the potential to provide a better and less expensive alternative for rehabilitation healthcare and may provide benefit to patients, physicians, and society through continuous monitoring in the ambulatory setting, early detection of abnormal conditions, supervised rehabilitation, and potential knowledge discovery through data mining of all gathered information.

Continuous monitoring with early detection likely has the potential to provide patients with an increased level of confidence, which in turn may improve quality of life. In addition, ambulatory monitoring will allow patients to engage in normal activities of daily life, rather than staying at home or close to specialized medical services. Last but not least, inclusion of continuous monitoring data into medical databases will allow integrated analysis of all data to optimize individualized care and provide knowledge discovery through integrated data mining. Indeed, with the current technological trend toward integration of processors and wireless interfaces, we will soon have coin-sized intelligent sensors. They will be applied as skin patches, seamlessly integrated into a personal monitoring system, and worn for extended periods of time.
